# Our Libel Suit

**Published:** 1855-12

**Authors:** 


*Our Libel Suit.*—We had hoped to have informed our readers, in this number, the result of the suit brought against us for libel, by J. D. Hill, of this city. This pleasure being denied us, (for the Journal will be mailed before the cause is reached) we hope that our delinquent subscribers, especially those easy souls who have favored us with their *patronage* for from three to ten years without contributing one cent to our finances, will immediately, upon the receipt of this admonition, forward to us our due. *That* being done we can pay our lawyers and any probable damages, with "a perfect looseness." Take heed, ye slow coaches! The Journal is now strong enough to lose a certain portion of its subscribers with profit to itself.

